# The innate immune response to coxsackievirus B3 predicts progression to cardiovascular disease and heart failure in male mice

**DOI:** 10.1186/2042-6410-2-2

**Published:** 2011-02-21

**Authors:** Jennifer A Onyimba, Michael J Coronado, Amanda E Garton, Joseph B Kim, Adriana Bucek, Djahida Bedja, Kathleen L Gabrielson, Tomas R Guilarte, DeLisa Fairweather

**Affiliations:** 1 Department of Environmental Health Sciences, Johns Hopkins University Bloomberg School of Public Health, Baltimore, MD 21205, USA; 2 Department of Pathology, Johns Hopkins University School of Medicine, Baltimore, MD 21205, USA; 3 Department of Environmental Health Sciences, Mailman School of Public Health, Columbia University, New York, NY 10032, USA

## Abstract

**Background:**

Men are at an increased risk of dying from heart failure caused by inflammatory heart diseases such as atherosclerosis, myocarditis and dilated cardiomyopathy (DCM). We previously showed that macrophages in the spleen are phenotypically distinct in male compared to female mice at 12 h after infection. This innate immune profile mirrors and predicts the cardiac immune response during acute myocarditis.

**Methods:**

In order to study sex differences in the innate immune response, five male and female BALB/c mice were infected intraperitoneally with coxsackievirus B3 (CVB3) or phosphate buffered saline and their spleens were harvested 12 h later for microarray analysis. Gene expression was determined using an Affymetrix Mouse Gene 1.0 ST Array. Significant gene changes were verified by quantitative real-time polymerase chain reaction or ELISA.

**Results:**

During the innate immune response to CVB3 infection, infected males had higher splenic expression of genes which are important in regulating the influx of cholesterol into macrophages, such as phospholipase A_2 _(PLA_2_) and the macrophage scavenger receptor compared to the infected females. We also observed a higher expression in infected males compared to infected females of squalene synthase, an enzyme used to generate cholesterol within cells, and Cyp2e1, an enzyme important in metabolizing cholesterol and steroids. Infected males also had decreased levels of the translocator protein 18 kDa (TSPO), which binds PLA_2 _and is the rate-limiting step for steroidogenesis, as well as decreased expression of the androgen receptor (AR), which indicates receptor activation. Gene differences were not due to increased viral replication, which was unaltered between sexes.

**Conclusions:**

We found that, compared to females, male mice had a greater splenic expression of genes which are important for cholesterol metabolism and activation of the AR at 12 h after infection. Activation of the AR has been linked to increased cardiac hypertrophy, atherosclerosis, myocarditis/DCM and heart failure in male mice and humans.

## Background

Cardiovascular disease (CVD) is the leading cause of death in the USA [1]. Heart failure can result from a number of cardiovascular conditions, including coronary artery disease, myocarditis and dilated cardiomyopathy (DCM), and men have a higher incidence and severity of these diseases than women [1-5]. Infections, such as coxsackievirus B3 (CVB3), toxins and hypersensitivity drug reactions are known to induce myocarditis [6,7]. Although myocarditis occurs more often in men, the rates of CVB3 infection are similar between men and women worldwide [8-10]. The factors that predict progression to myocarditis and DCM remain unknown.

We have developed a mouse model of autoimmune myocarditis and DCM using a heart-passaged strain of CVB3 that includes infectious virus and heart antigens in the inoculum [11]. All infected mouse strains develop acute myocarditis from day 8 to 12 post infection (pi), but only certain strains such as A/J and BALB/c develop DCM by day 35 pi [11]. Male BALB/c mice infected with heart-passaged CVB3 develop more severe acute myocarditis than females. More severe disease in males is associated with increased Toll-like receptor (TLR)4 expression on mast cells and macrophages in the heart during acute myocarditis and in the spleen at 12 h pi [12-14]. TLR4 signalling following infection increases cardiac interleukin (IL)-1β and IL-18 resulting in a more prominent T helper type 1 (Th1) immune response in males [12,13,15]. Although males have more severe CVB3 myocarditis, the virus replicates to the same level in the hearts of both male and female mice [12,13]. Gonadectomy of male BALB/c mice reduces myocarditis, making males appear immunologically like females with increased IL-4 and more alternatively activated macrophages and regulatory T cells in the heart [14]. Furthermore, as early as 6 h-12 h after infection mast cells and macrophages obtained from the spleen or peritoneum of males express more TLR4 than females, displaying the same innate immunological profile in the spleen as observed in the heart during acute myocarditis [13].

As innate immunity directs the adaptive immune response [13,16], we hypothesized that sex differences in innate immune genes in the spleen would uncover pathways that are important in the susceptibility to acute myocarditis and DCM. We found that the primary gene networks elevated in males compared to females at 12 h after infection involved cholesterol metabolism and activation of the androgen receptor (AR) in immune cells.

## Methods

### Mice

BALB/cJ (BALB/c) mice were obtained from The Jackson Laboratory (ME, USA). Mice were maintained under pathogen-free conditions in the animal facility at the Johns Hopkins School of Medicine and approval was obtained from the Animal Care and Use Committee of the Johns Hopkins University for all procedures.

### Inoculations

Male and female BALB/c mice (8 to 10 weeks old) were inoculated intraperitoneally (ip) with 10^3 ^plaque forming units (PFU) of heart-passaged CVB3 (contains infectious virus and heart tissue) diluted in sterile phosphate buffered saline (PBS) and the spleen, pancreas and sera harvested at 12 h and 48 h pi. Age matched control males and females received PBS only. In separate experiments, mice received CVB3 on day 0 and echocardiography was conducted at day 10 pi and hearts were collected for histology at day 12 and 35 pi. CVB3 (Nancy strain) was obtained from the American Type Culture Collection (ATCC, VA, USA), grown in Vero cells (ATCC) and passaged through the heart as described [11]. Mice inoculated ip with uninfected cardiac tissue supernatant diluted in PBS, or PBS alone, do not develop myocarditis [12-14]. For this reason PBS alone injections were used as uninfected controls for the innate experiments.

### Myocarditis and DCM

Hearts were fixed in 10% buffered formalin, stained with haematoxylin and eosin (H&E) and myocarditis was assessed as the percentage of the heart section with inflammation compared to the overall size of the heart section using a microscope eyepiece grid. The sections were examined by two independent investigators. The development of DCM was assessed by gross observation of histology sections at low magnification and functionally by echocardiography. Individual experiments were conducted three times with 7 - 10 mice per group.

### Echocardiography

Cardiac function was examined by trans-thoracic echocardiography in conscious mice using the Sequoia Acuson C256 ultrasound machine (PA, USA) equipped with a 15 MHz linear transducer, as previously described [17]. The heart was imaged in a two-dimensional mode in the parasternal short axis view. From the M mode, the left ventricular (LV) wall thickness and chamber dimensions were determined. Ejection fraction represents stroke volume (the volume of blood ejected with each beat) divided by end diastolic LV volume.

### Plaque assay

The spleen and pancreas from individual mice were homogenized at 10% weight/volume in 2% minimal essential medium (MEM) and individual supernatants were used in plaque assays to determine the level of infectious virus [11]. Virus titres are expressed as the mean plaque-forming unit (PFU)/g tissue ± standard error of mean (SEM) and the limit of detection is 10 PFU/g of tissue. Individual experiments were conducted three times with 5 - 7 mice per group.

### ELISA

Spleens were homogenized at 10% weight/volume in 2% MEM and individual supernatants used in ELISA (seven/group). Secreted PLA_2 _levels were determined in homogenized supernatants using USCN Life Science kits (Wuhan, China), according to the manufacturer's instructions. PLA_2 _levels were expressed as pg/g of spleen tissue ±SEM. The kit did not specify sPLA_2 _subtype.

### RNA extraction and microarray

Individual spleens from uninfected PBS-inoculated control and heart-passaged CVB3-infected male and female BALB/c mice were used for microarray analysis (five/group, not pooled) or in a separate experiment to verify microarray data by real-time polymerase chain reaction (RT-PCR; seven/group, not pooled). Spleens were harvested at 12 h pi, flash frozen in liquid nitrogen and stored at -80°C. The spleens were homogenized in 2 mL TRIzol (Invitrogen, CA, USA) according to the manufacturer's protocol. The PureLink Micro-to-Midi Total RNA Purification System (Invitrogen) was used for extraction and purification of RNA. RNA was quantified using a NanoDrop spectrophotometer and quality assessed by RNA Nano LabChip analysis on an Agilent BioAnalyzer 2100 (Agilent Technologies, CA, USA). Processing and GeneChip analysis for microarray were performed on five samples for each treatment group. We processed 100 ng of the total RNA for hybridization to Affymetrix Mouse Gene ST 1.0 microarrays using the Affymetrix GeneChip Whole Transcript Sense Target Labeling Assay, according to the manufacturer's protocol and previously published methods (Affymetrix, CA, USA) [18,19]. The Affymetrix Mouse GeneChip Gene 1.0 ST Array interrogates 28,853 well-annotated genes with 764,885 distinct probes. The expertise, facilities and instrumentation for Affymetrix GeneChip experimentation and analyses were provided and supported by the Johns Hopkins Malaria Research Institute.

Analysis of microarray data was performed with Partek Genomics Suite (GS) Version 6.4 (Partek, MO, USA). Gene expression patterns for each gene were normalized to the median array intensity for all chips and data from infected animals was normalized to uninfected PBS controls [18]. Microarray data were analysed with Partek GS software by 2-way ANOVA in order to look for significant differences between conditions (with sex and infection as factors) and then *P *values and fold changes were generated using Fisher's least significant difference (LSD) post hoc analysis for comparisons of sex. False discovery rate (FDR) corrections for multiple comparisons (Benjamini-Hochberg) were applied to reduce the total number of false-positives. Genes were considered significant if they had a *P *value less than 0.05. Ingenuity Pathway Analysis (IPA; Ingenuity Systems, CA, USA) was used to generate IPA network data by inputting microarray data analysed with Partek GS. Ingenuity generates networks by identifying published gene relationships and provides an IPA score (*P *value) indicating the likelihood that the significantly up or down-regulated genes found in our microarray would be present in a given network. The *P *value is calculated as the -log of the Fisher's exact test.

### Microarray data accession number

The Affymetrix gene expression data were deposited to the GEO http://www.ncbi.nlm.nih.gov/geo with accession number GSE26630.

### Validation of microarray data by quantitative RT-PCR

Results obtained from microarray data were verified in a separate experiment using seven mice/group. Total RNA from spleens was validated by qRT-PCR using Assay-on-Demand primers and probe sets and the ABI 7000 Taqman System from Applied Biosystems (CA, USA), according to Rangasamy *et al*. [20]. Hypoxanthine phosphoribosyltransferase 1 (HPRT) was used as a normalization control. There was no significant difference in HPRT expression in the spleen between males and females before or after infection. The mRNA data are presented as a relative gene expression (RGE). RGE is calculated as the ratio of target gene expression [fold change of messenger RNA (mRNA) of interest) to the normalization control gene expression (fold change of normalization control mRNA). Data is expressed as the mean of seven mice/group ± SEM. Normally distributed data were analysed by the Student's *t *test. The Mann-Whitney *U *test was used to evaluate nonparametric data. A value of *P *< 0.05 was considered significant.

### Statistical analysis

Statistical analysis of the microarray data is described in the 'RNA Extraction and Microarray' section of the Methods. All other data were analysed by the Student's *t *test for normally distributed data and the Mann-Whitney *U *test for nonparametric data. A value of *P *< 0.05 was considered significant.

## Results

### Males develop increased myocarditis and progress to heart failure and DCM

**Figure 1:**
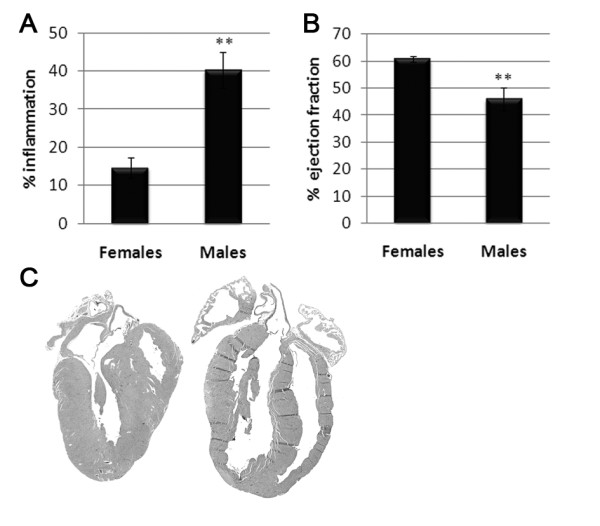
**Males develop increased myocarditis and progress to heart failure and myocarditis and dilated cardiomyopathy (DCM)**. Male and female BALB/c mice were inoculated intraperitoneally with 10^3 ^plaque forming units of heart passaged coxsackievirus B3 on day 0 and (A) myocarditis assessed histologically at day 12 post infection (pi) and (B) heart function (% ejection fraction) assessed by echocardiography at day 10 pi. An ejection fraction ≤45% indicates heart failure. (C) Dilation is observed in male hearts (right) but not in female hearts (left) in histology sections at day 35 pi. Magnification × 5. DCM was confirmed in males but not females at day 90 pi by echocardiography (data not shown). Data show the mean ± standard error of the mean of 7 - 10 mice per group. **, *P *< 0.01.

We showed previously that male BALB/c mice with acute CVB3 myocarditis develop significantly increased inflammation in the heart compared to females, while the virus replicates in the heart at the same level in both sexes [12,13]. Increased acute CVB3 myocarditis in males- compared to females (Figure 1A) was associated with reduced heart function with a lower ejection fraction by echocardiography at day 10 pi (Figure 1B). An ejection fraction ≤45% is indicative of heart failure [21]. By day 35 pi male hearts became dilated while female hearts appeared normal (Figure 1C).

### Genes altered in male versus female spleens prior to and following infection

Previously, we showed that immune factors that determine sex differences in CVB3 myocarditis, such as increased TLR4 expression on mast cells and macrophages following CVB3 infection, are upregulated as early as 12 h pi in the spleen of male compared to female mice [13,22]. As the innate immune response in the spleen is important in driving the adaptive immune response, we examined sex differences in gene expression by microarray at 12 h in the spleens of male and female BALB/c mice inoculated with 10^3 ^PFU CVB3 or PBS ip on day 0. Although we conducted a two-way ANOVA analysis of data from the four groups (males versus females, PBS versus CVB3), in this paper we report only the effect of sex (we do not report gene differences between PBS versus CVB3 infected groups).

After the two-way ANOVA, we performed a post-hoc Fisher's LSD analysis in order to identify gene changes between uninfected males and uninfected females or infected males and infected females with a *P *< 0.05 (Table 1). FDR corrections were applied in order to reduce the total number of false-positives. The main category of genes that was identified following FDR analysis in infected or uninfected male versus female spleens was × and Y-linked genes (Table 1). These × and Y-linked genes are routinely observed in microarray studies of sex differences in hearts from humans and mice [23].

**Table 1:** Genes altered in the spleen of males compared to females using false discovery rate analysis.

GenBank	Gene	Gene name	Fold increase	*P *value
*Higher expression in uninfected males*				
NM_012011	Eif2s3y	Eukaryotic translation initiation factor 2	53.88	3.3 × 10^-22^
NM_012008	Ddx3y	DEAD box polypeptide, Y-linked	38.95	1.8 × 10^-20^
NM_009484	Uty	Ubiquitously transcribed tetratricopeptide repeat gene	23.87	2.3 × 10^-17^
NM_011419	Jarid1d	Jumonji, AT rich interactive domain	12.21	3.5 × 10^-16^
				
*Lower expression in uninfected males*				
NR_001463	Xist	Inactive X-specific transcripts	-64.65	4.2 × 10^-20^
NM_009483	Utx	Ubiquitously transcribed tetratricopeptide repeat gene	-1.45	1.7 × 10^-7^
NM_012011	Eif2s3x	Eukaryotic transl. initiation factor 2	-1.33	1.2 × 10^-5^
				
*Higher expression in infected males*				
NM_012011	Eif2s3y	Eukaryotic transl. initiation factor 2	51.01	4.1 × 10^-22^
NM_012008	Ddx3y	DEAD box polypeptide, Y-linked	39.24	1.7 × 10^-20^
NM_009484	Uty	Ubiquitously transcribed tetratricopeptide repeat gene	24.99	1.8 × 10^-17^
NM_011419	Jarid1d	Jumonji, AT rich interactive domain	12.21	3.5 × 10^-16^
				
*Lower expression in infected males*				
NR_001463	Xist	Inactive X-specific transcripts	-62.49	4.8 × 10^-20^
NM_009483	Utx	Ubiquitously transcribed tetratricopeptide repeat gene	-1.64	3.8 × 10^-9^
NM_012010	Eif2s3x	Eukaryotic translation initiation factor 2	-1.33	1.1 × 10^-5^
NM_011861	Pacsin1	Protein kinase C, casein kinase	-1.24	2.1 × 10^-6^

### Genes increased in male versus female spleens prior to infection

As our previous research has shown that distinct immune differences exist between infected males and infected females at 12 h pi in the spleen that relate to heart disease, we analysed the microarray data without correcting for multiple comparisons. The primary problem with this type of analysis is that it is likely to generate false positives. Thus, findings must be verified with other methods such as RT-PCR or ELISA. We suspect that small gene changes will be important indicators of disease susceptibility because the innate immune response to CVB3 between males and females is very similar, differing mainly in amplitude (severity) [12-14]. For example, both male and female splenocytes upregulate TLR4 following CVB3 infection but more immune cells express TLR4 in males than in females [13]. Genes found to be increased in uninfected males versus uninfected females are listed in Table 2.

**Table 2:** Genes with greater expression in the spleen of males compared to females prior to infection without correction for multiple comparisons.

GenBank	Gene	Gene name	Fold increase	*P *value
NM_009830	Ccne2	Cyclin E2	1.90	0.008
NM_021282	Cyp2e1	Cytochrome P450 2e1	1.80	9.4 × 10^-4^
NM_007606	Ca3	Carbonic anhydrase 3	1.78	0.01
NM_011921	Aldh1a7	Aldehyde dehydrogenase family 1a7	1.68	0.03
NM_023135	Sult1e1	Sulphotransferase family 1e1	1.65	0.002
NM_007691	Chek1	Checkpoint kinase 1	1.64	0.03
NM_001012273	Birc5	Baculoviral IAP/Survivin	1.63	0.04
NM_020504	Cldn13	Claudin 13	1.62	0.03
NM_025569	Mgst3	Microsomal glutathione S-transferase 3	1.61	0.04
NM_010360	Gstm5	Glutathione S-transferase, mu5	1.59	0.04
NM_008253	Hmgb3	High mobility group box 3	1.56	0.03
NM_017370	Hp	Haptoglobin	1.53	0.03
NM_172479	Slc38a5	Solute carrier family 38, member 5	1.53	0.04
NM_177744	Apol10a	Apolipoprotein L 10a	1.53	0.02
NM_028039	Esco2	Establishment of cohesion 1 homolog 2	1.53	0.04
NM_008613	Mns1	Meiosis-specific nuclear structural protein	1.52	0.04
NM_009479	Uros	Uroporphyrinogen III synthase	1.51	0.03
NM_029522	Gpsm2	G-protein signalling modulator 2	1.50	0.04
NM_013467	Aldh1a1	Aldehyde dehydrogenase 1a1	1.49	0.03
NM_010480	Hsp90aa1	Heat shock protein 90kDa alpha	1.19	0.04

**Figure 2:**
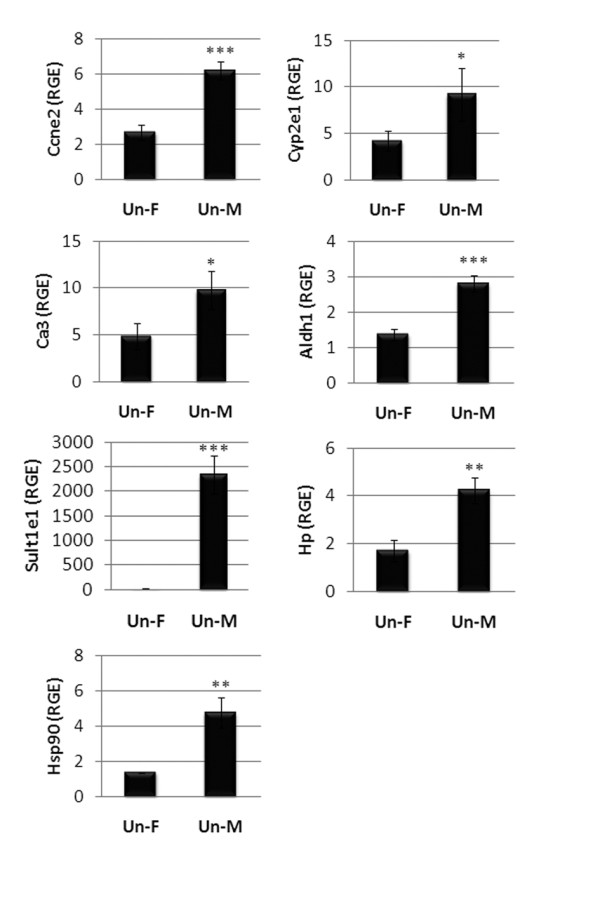
**Verification of genes increased in males compared to females prior to infection**. Gene changes between uninfected males (Un-M) and uninfected females (Un-F) in Table 2 were verified by quantitative real-time polymerase chain reaction. Genes increased in males included: cyclin E2 (Ccne2); Cyp2e1; carbonic anhydrase 3 (Ca3); aldehyde dehydrogenase (Aldh1a7); sulphotransferase1e1 (Sult1e1); haptoglobin (Hp); and heat shock protein 90 (Hsp90aa1). Relative gene expression (RGE) normalized to hypoxanthine phosphoribosyltransferase (HPRT) is shown as the mean ± standard error of the mean of seven mice per group. *, *P *< 0.05; **, *P *< 0.01; ***, *P *< 0.001.

Using mRNA obtained in a separate experiment from the one used for the microarray analysis, we verified by qRT-PCR that the top five genes listed in Table 2 were more highly expressed in uninfected males compared to uninfected females (Table 2, Figure 2). Several of these genes are important in regulating oxidative stress to infection including Cyp2e1, aldehyde dehydrogenase (Aldh1a7) and sulphotransferase 1e1 (Sult1e1; Figure 2). The cytochrome P450 Cyp2e1 is known to be elevated in men and is important in metabolizing cholesterol and steroids. Cyp2e1 also induces the production of reactive oxygen species (ROS) that activate proinflammatory responses and reduce viral replication [24,25]. Several other genes reported to be important in promoting heart disease and regulating lipid metabolism were elevated in the spleen of uninfected males compared to uninfected females (Table 2). These genes included: carbonic anhydrase 3 (Ca3), an enzyme known to contribute to cardiac hypertrophy and heart failure when expressed in the heart [26]; haptoglobin (Hp), a biomarker of inflammation and cardiovascular disease (CVD) [27]; and heat shock protein 90 (Hsp90aa1; Table 2). We verified that these genes had a higher expression in uninfected males by qRT-PCR even for Hsp90, which had a fold change of only 1.19 (Table 2, Figure 2). We did not verify all of the genes identified in Table 2 and so other genes in this list could include false positives. Our findings suggest that uninfected males have an underlying immune profile in the spleen that may predispose them to a proinflammatory response and inflammatory heart disease following infection.

### Endocrine system development and function genes increase in CVB3 infected males compared to infected females at 12 h pi

**Figure 3:**
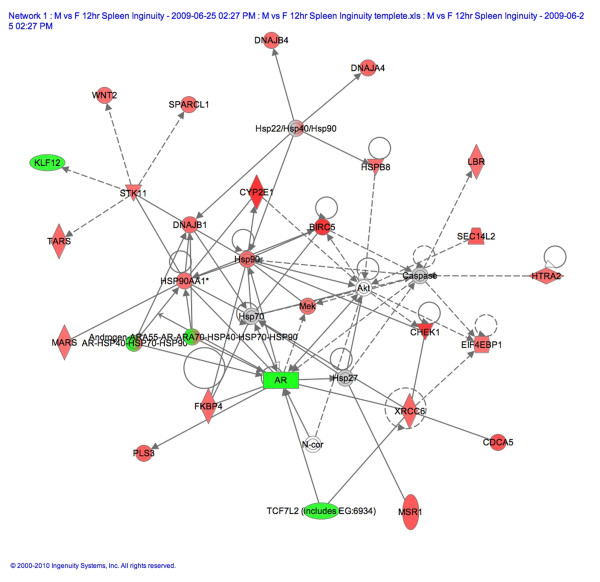
**Endocrine system development and function genes increase in the spleen of CVB3 infected males at 12 h pi**. Male and female BALB/c mice were inoculated intraperitoneally with 10^3 ^plaque forming units of coxsackievirus B3 (CVB3) or phosphate buffered saline on day 0 and microarray was conducted on individual spleens at 12 h post infection (pi) (five/group). Ingenuity Pathway Analysis of microarray data that had not been corrected for multiple comparisons revealed that the top gene network in males involved endocrine system development and function (Network 1). Red and green represents genes significantly up- or down-regulated in CVB3 infected males compared to infected females, respectively.

**Figure 4:**
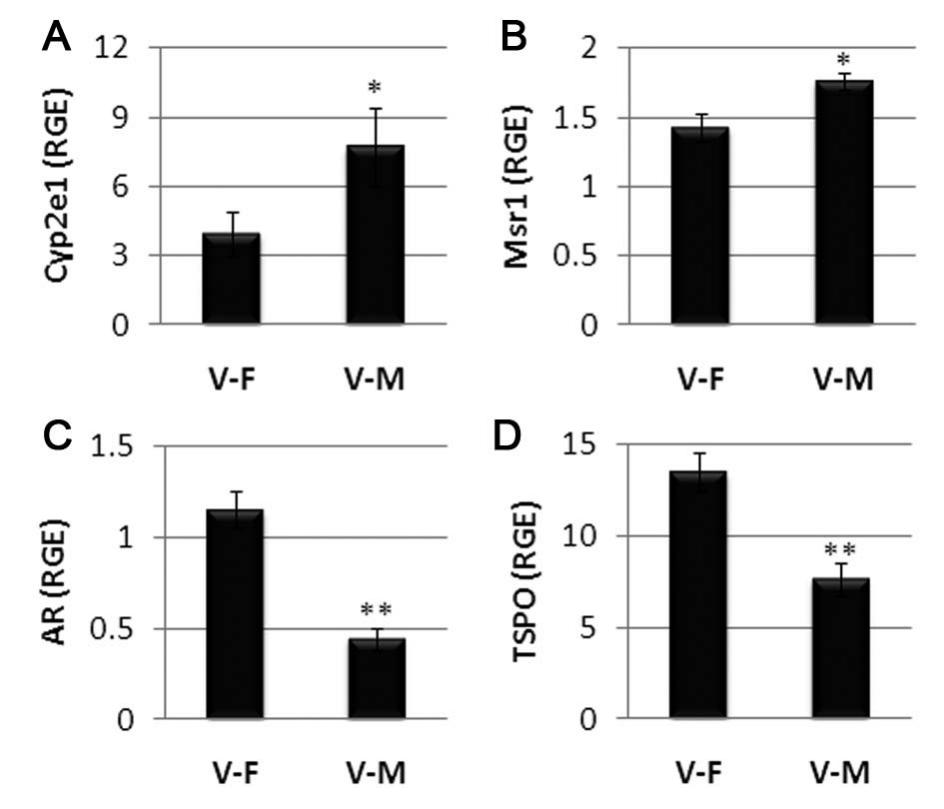
**Verification of endocrine system development and function genes**. Using a separate experiment from the one used for the microarray, gene changes in Figure 3 were verified by quantitative real-time polymerase chain reaction between coxsackievirus B3 infected males (V-M) and infected females (V-F). Verified genes included: (A) Cyp2e1; (B) the macrophage scavenger receptor (Msr1); (C) the androgen receptor (AR); and (D) translocator protein 18 kDa (TSPO). Relative gene expression (RGE) normalized to hypoxanthine phosphoribosyltransferase (HPRT) is shown as the mean ± standard error of the mean of seven mice per group. *, *P *< 0.05; **, *P *< 0.01.

Analysis of the microarray data, without correcting for multiple comparisons, revealed a number of genes expressed more highly in CVB3 infected males than in infected females (data not shown). IPA of the data found that the gene network with the most genes present, and thus the highest IPA score, from our study of the spleen of infected males compared to infected females involved endocrine system development and function with an IPA score of 42 (*P *= 1 × 10^-42^; Figure 3). The IPA score (*P *value) indicates the likelihood that the significantly up or down-regulated genes found in our microarray would be present in a given gene network. Using mRNA obtained in a separate experiment from the one used for the microarray analysis, we verified by qRT-PCR several of the genes shown in Figure 3. Cyp2e1 was the most highly expressed gene in this network with a fold increase of 1.8 (*P *= 9.4 × 10^-4^; Figure 3). Cytochrome P450 s such as aromatase play a critical role in the metabolism of steroids [28]. We were surprised to find that the AR was downregulated in the spleen of CVB3 infected males compared to infected females (Figure 3). However, it has been reported that elevated levels of testosterone decrease mRNA expression of the AR but increase AR protein and indicate activation or the use of the receptor [28,29]. Hsp90 acts as a chaperone preventing movement of the AR to the nucleus. We found that Hsp90 mRNA was more highly expressed in males prior to infection (Table 2, Figure 2). We verified that Cyp2e1 (Figure 3 and 4A) and the macrophage scavenger receptor (Msr1; Figures 3 and 4B), which transports oxidized lipids into macrophages, had greater expression in CVB3 infected males than infected females at 12 h pi. Reduced mRNA expression of the AR in CVB3 infected males compared to infected females was also verified by qRT-PCR (Figure 4C). TSPO is the rate-limiting step for steroid synthesis and is expressed in mast cells, macrophages, heart tissue and the spleen, for example [30]. We found that, similar to the AR, TSPO mRNA levels were also decreased in infected males compared to infected females at 12 h pi in the spleen (Figure 4D). We did not verify other genes from this network and it is likely that some of the genes shown in Figure 3 are false positives. However, we verified that Msr1, TSPO, AR and Cyp2e1, genes critical for cholesterol and steroid synthesis, are more elevated or 'activated' in the spleen of infected males compared to infected females at 12 h pi.

### CVB3 replicates at the same level in females and males during the innate immune response

**Figure 5:**
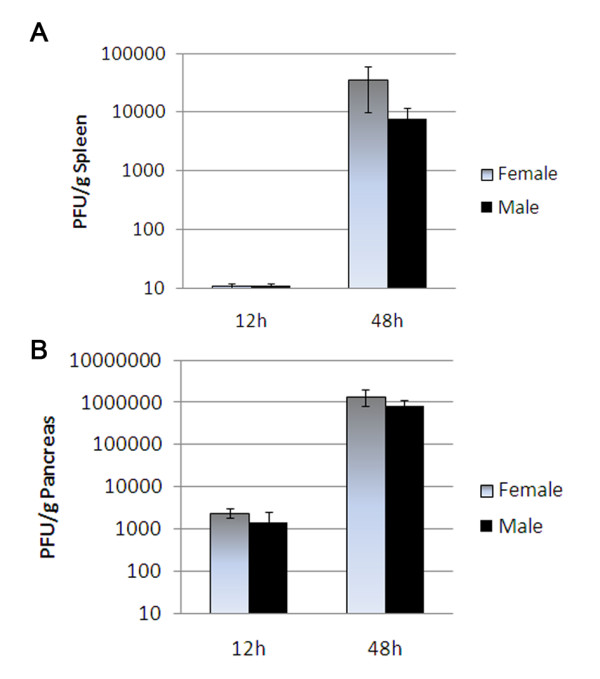
**CVB3 replicates at the same level in males and females during the innate immune response**. Male and female BALB/c mice were inoculated intraperitoneally with 10^3 ^plaque forming units (PFU) of coxsackievirus B3 (CVB3)or phosphate buffered saline (PBS) on day 0 and the level of viral replication in the spleen (A) and pancreas (B) examined at 12 h and 48 h post infection. No viral replication was detected in uninfected PBS controls. No significant differences in viral replication were observed in CVB3 infected males compared to infected females. Data show the mean ± standard error of the mean of 5 - 7 mice pergroup.

In order to examine whether sex differences in gene expression in CVB3 infected males and infected females were related to viral replication, we inoculated male and female BALB/c mice with 10^3 ^PFU CVB3 or PBS ip on day 0 and examined the level of viral replication in the spleen and pancreas at 12 and 48 h pi (Figure 5). No viral replication was detected in uninfected PBS controls (data not shown) or at 12 h in the spleen of infected mice (Figure 5A), confirming earlier findings [22]. There was no significant difference in viral replication between CVB3 infected males and infected females at 12 h pi in the pancreas or at 48 h pi in the spleen and pancreas (the pancreas is a target organ for CVB3 infection; Figure 5A and 5B). Thus, altered gene expression that occurs between males and females at 12 h pi in the spleen by microarray is not simply due to differences in viral replication.

### Genes associated with cardiovascular disease increase in CVB3 infected males compared to infected females at 12 h pi

The network with the second highest number of genes, and thus second highest IPA score, identified by IPA of data that had not been corrected for multiple comparisons in infected males compared to infected females was DNA replication, recombination and repair, with an IPA score of 41 (*P *= 1 × 10^-41^; data not shown). Remember, the IPA score (*P *value) indicates the likelihood that the significantly up or down-regulated genes found in our microarray would be present in a given gene network. This gene network also received the highest IPA score for data that had not been corrected for multiple comparisons between uninfected males and uninfected females, with an IPA score of 46 (*P *= 1 × 10^-46^; data not shown). None of the other networks comparing uninfected males to uninfected females corresponded to those revealed for CVB3 infected males versus infected females, which indicates that infection activated a distinct immune profile in the spleen of males that was not simply due to baseline sex differences.

**Figure 6:**
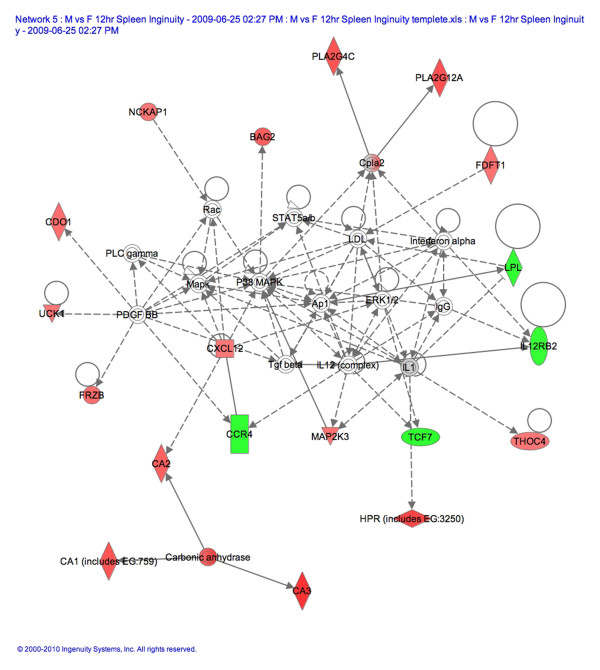
**Cardiovascular disease genes increase in the spleen of CVB3 infected males at 12 h pi**. Male and female BALB/c mice were inoculated intraperitoneally with 10^3 ^plaque forming units of coxsackievirus B3 (CVB3) or phosphate buffered saline on day 0 and a microarray conducted on individual spleens at 12 h pi (five/group). Ingenuity Pathway Analysis of microarray data that had not been corrected for multiple comparisons revealed that genes associated with cardiovascular disease were increased in CVB3 infected males compared to infected females (Network 5). Red and green represents genes significantly up- or down-regulated, respectively.

Unexpectedly, we found that the networks with the 3rd and 5th highest IPA scores in the spleen of CVB3 infected males compared to those in the infected females from data that had not been corrected for multiple comparisons included cardiovascular system development and function with an IPA score of 38 (*P *= 1 × 10^-38^; data not shown) and cardiovascular and metabolic disease with an IPA score of 29 (*P *= 1 × 10^-29^; Figure 6, Table 3). These CVD gene networks were not observed in uninfected males versus uninfected females. Genes with well known roles in promoting CVD that were more highly expressed in the spleen of CVB3 infected males compared to infected females at 12 h pi included carbonic anhydrase (Ca1-Ca3; Figure 6, Table 2 and 3) and PLA_2 _(Cpla2, Pla2g4c, Pla2g12a; Figure 6, Table 3) [26]. Elevated lipoprotein-associated (Lp)-PLA_2 _is associated with the development of atherosclerosis and stroke [31]. Other key proteins in this network included: farnesyl-diphosphate faresyltranserferase 1 (Fdft1), also called squalene synthase, which increases intracellular cholesterol levels [32]; MAP2 kinase 3 (Map2k3) which has been shown to contribute to sex-based differences in myocardial remodelling and heart failure [33,34]; and the chemokine CXCL12 which regulates neutrophil and macrophage function (Figure 6, Table 3) [35].

**Table 3:** Cardiovascular disease, metabolic disease, and genetic disorder genes (Network 5).

GenBank	Gene	Gene name	Fold increase	*P *value
*Higher expression in males*				
NM_007606	Ca3	Carbonic anhydrase 3	1.78	0.01
NM_183423	Pla2g12a	Phospholipase A2, group XIIA	1.43	0.02
NM_001004762	Pla2g4c	Phospholipase A2, group IVC	1.40	0.02
NM_145392	Bag2	BCL2-associated athanogene	1.32	0.02
NM_009801	Ca2	Carbonic anhydrase 2	1.31	0.03
NM_011356	Frzb	Frizzled-related protein	1.20	0.02
NM_033037	Cdo1	Cysteine deoxygenase 1	1.19	0.01
NM_010191	Fdft1	Farnesyl-diphosphate farnesyltransferase 1	1.18	0.02
NM_008928	Map2k3	MAP2 kinase 3	1.15	0.03
NM_011568	Thoc4	THO complex 4	1.13	0.04
NM_011675	Uck1	Uridine-cytidine kinase 1	1.13	0.04
NM_001012477	Cxcl12	Chemokine ligand 12	1.12	0.005
NM_016965	Nckap1	NCK-associated protein 1	1.12	0.04
				
*Lower expression in males*				
NM_009331	Tcf7	Transcr. factor 7, T cell-specific	-1.33	0.02
NM_009916	Ccr4	Chemokine receptor 4	-1.31	0.0002
NM_008509	Lpl	Lipoprotein lipase	-1.31	0.005
NM_008354	Il12rb2	Interleukin 12 receptor, beta 2	-1.28	0.01

**Figure 7:**
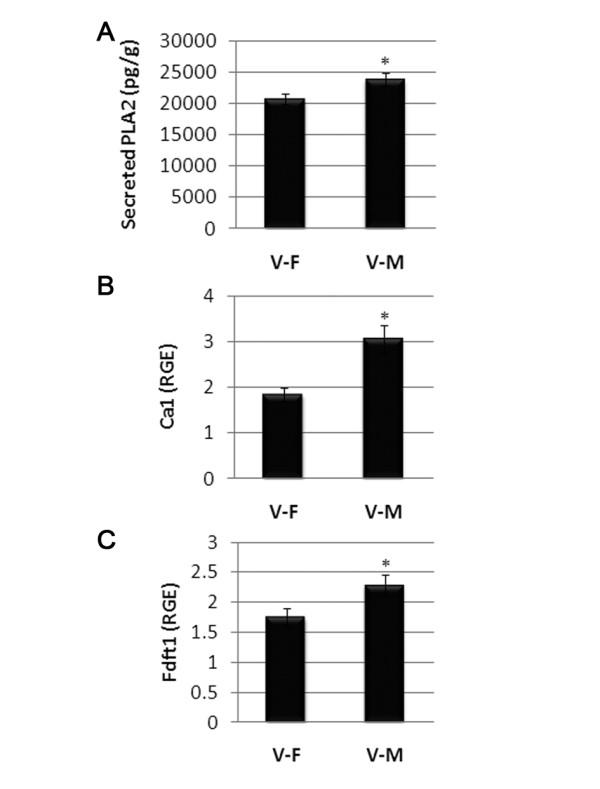
**Verification of cardiovascular disease genes in infected males**. Using separate experiments from the one used for the microarray, gene changes in Table 3 were verified by ELISA or quantitative real-time polymerase chain reaction (qRT-PCR) between coxsackievirus B3 - infected males (V-M) and infected females (V-F). Secreted phospolipase A_2 _(PLA2) was verified in the spleen at (A) 48 h post infection (pi) by ELISA, while (B) carbonic anhydrase 1 (Ca1) and (C) squalene synthase (Fdft1) were verified by qRT-PCR at 12 h pi. Relative gene expression (RGE) normalized to hypoxanthine phosphoribosyltransferase is shown as the mean ± standard error of the mean of seven mice per group. *, *P *< 0.05.

Using separate experiments from the one used for the microarray, we verified by qRT-PCR or ELISA several of the genes shown in Table 3. We confirmed that PLA_2 _was increased in the spleen of CVB3 infected males versus infected females using ELISA (Figure 7A) and that infected males had greater mRNA expression of carbonic anhydrase 1 (Ca1; Figure 7B) and squalene synthase (Fdft1; Figure 7C) by qRT-PCR. Although we were able to confirm that carbonic anhydrase 3 (Ca3) expression was greater in uninfected males than uninfected females (Figure 2), we were unable to confirm that Ca3 was elevated in CVB3 infected males by RT-PCR. We did not verify other genes in Table 3 and so the list could include other false positives. However, we were able to verify that Ca1, PLA_2 _and squalene synthase had higher expression in the spleen of CVB3 infected males than in infected females at 12 h pi (Figure 7). Thus, a number of genes associated with the development of CVD and heart failure, and genes that regulate cholesterol metabolism, show greater expression in the spleen of CVB3 infected males during innate immunity. Our findings indicate that these genes may be involved in initiating the immune response that results in inflammatory heart disease. Further studies are necessary to confirm their role.

## Discussion

**Figure 8:**
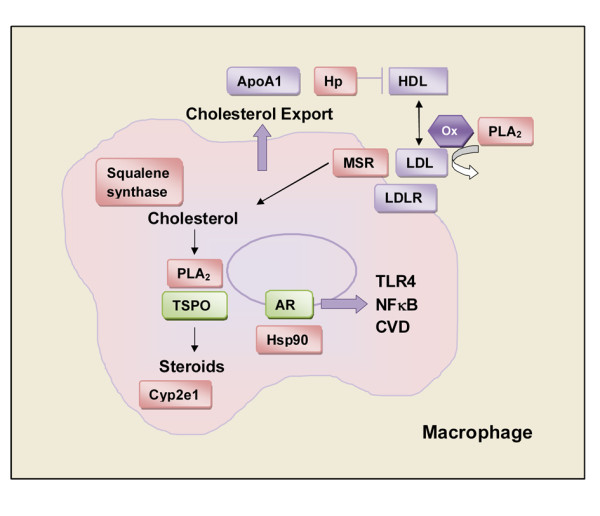
**Genes increased in males involve regulation of cholesterol metabolism and steroidogenesis**. In this study we verified that a number of genes that are important in regulating cholesterol influx into cells and steroidogenesis are more highly expressed in the spleen of males prior to, or during, the innate immune response to coxsackievirus B3 (CVB3) infection. Genes with a higher expression in males are shown in pink boxes, genes with lower expression in males in green boxes and genes we did not examine in purple boxes. We suggest the following hypothesis as one possible scenario leading to increased inflammation in males. Haptoglobin (Hp) is known to bind Apo-A1 preventing the conversion of proinflammatory low-density lipoproteins (LDLs) to anti-inflammatory high-density lipoproteins (HDLs), thereby increasing the LDL levels. Elevated phospholipase A_2 _(PLA_2_) in males may facilitate the oxidation (Ox) of LDL allowing it to bind the macrophage scavenger receptor (Msr1/MSR) and import LDL-cholesterol into immune cells, particularly macrophages. Increased levels of squalene synthase in males may facilitate intracellular cholesterol synthesis in immune cells. PLA_2 _is an endogenous ligand for translocator protein 18 kDa (TSPO), the rate-limiting step for steroidogenesis within cells. Cyp2e1 participates in the metabolism of cholesterol and steroids and contributes to an increased proinflammatory response in males. Increased production of androgens within macrophages allows the androgen receptor (AR) to release from its chaperone Hsp90 and move to the nucleus to stimulate the proinflammatory immune response associated with increased myocarditis and heart failure in males following CVB3 infection.

Recent evidence indicates that the innate immune response is critical in determining the adaptive response to infection or vaccination. We previously showed that as early as 12 h pi males respond to CVB3 infection with an elevated proinflammatory response [13]. Elevated TLR4 expression on mast cells and macrophages in the spleen and heart of CVB3 infected males not only increases acute myocarditis but induces expression of the profibrotic cytokine IL-1β resulting in cardiac dilatation and heart failure later [13-15,36]. In this study, we examined the sex differences in gene expression in the spleen of BALB/c mice prior to, and during, the innate immune response to CVB3 infection. We found that FDR analysis of sex differences following infection revealed mainly differences in × and Y-linked genes. However, by analysing the microarray data without correcting for multiple comparisons we were able to identify by Partek and Ingenuity software and to verify by qRT-PCR and ELISA a number of other gene changes between sexes. Similar to our findings, several studies in rodents and humans examining sex differences in heart disease using FDR analysis yielded few gene changes aside from × and Y-linked genes [4,23,34,37]. This is an important issue for the study of sex differences in heart disease and other chronic inflammatory conditions. These findings suggest that small gene changes are likely to be important in driving sex differences in inflammatory diseases. Most of the low-fold gene changes we verified from the IPA networks involved genes critical to the regulation of cholesterol and steroid synthesis in immune cells and in macrophages in particular (Figure 8).

For many years it has been known that underlying sex differences exist in the expression of hepatic enzymes such as cytochrome P450 s, sulphotransferases and glutathione *S*-transferases [24,38]. We verified that male spleens have a higher expression of Cyp2e1 and Sult1e1 (Table 2, Figures 2 and 4). These enzymes are important in metabolizing drugs, steroids, fatty acids and environmental chemicals, as well as amplifying a protective immune response against infection. Thus, elevated levels of these enzymes in the spleen of males may predispose them to increased inflammation following CVB3 infection.

In addition, we found that males respond to CVB3 infection by upregulating genes specifically involved in cholesterol metabolism (Figure 8). Squalene synthase (Fdft1) regulates intracellular cholesterol levels particularly the endogenous production of steroid hormones, vitamin D, bile acids and lipoprotein particles (Figures 6, 7 and 8, Table 3). Squalene synthase inhibitors have been found to reduce plasma levels of total- and low-density lipoprotein (LDL)-cholesterol in clinical studies [32]. Another source of cholesterol for macrophages comes from extracellular LDL-cholesterol. LDL is recruited from the circulation to the cell wall where it becomes oxidized by secreted PLA_2 _(sPLA_2_) among other factors [39]. The superfamily of PLA_2 _enzymes includes 15 distinct groups that fall into four main categories: secreted (sPLA_2_); cytosolic (cPLA_2_); calcium-independent PLA_2_; and Lp-PLA_2 _[40]. Approximately 80% of Lp-PLA_2 _is associated with LDL in the sera, and elevated levels of Lp-PLA_2 _and sPLA_2_s occur in patients with atherosclerosis [31,40,41]. PLA_2 _catalyzes the removal of fatty acids from the *sn-*2 position of membrane phospholipids releasing arachidonic acid for the generation of lipid mediators that are crucial for many inflammatory processes such as leukotrienes, prostaglandins and thromboxanes [42,43]. Increased sPLA_2 _levels have been associated with a number of inflammatory conditions including: rheumatoid arthritis; sepsis; psoriasis; pancreatitis; and cancer [43,44]. cPLA_2 _levels increase in phagocytic cells, such as mast cells, macrophages and neutrophils, following their activation where cPLA_2 _has been found to be essential for phagocytic function [42]. Similarly, sPLA_2 _is secreted from mast cells and macrophages following activation. In this study we were able to verify increased sPLA2 in the spleen of CVB3 infected males compared to infected females (Figures 6 and 7A, Table 3). PLA_2 _is also a ligand for TSPO and so elevated levels of PLA_2 _in the spleen of males could facilitate AR-mediated activation of immune cells during the innate immune response to CVB3 infection (Figure 8). Oxidized-LDL becomes a ligand for LDL receptors (LDLR) and macrophage scavenger receptors (Msr1) that transport cholesterol into macrophages to be metabolized to steroids and other lipid mediators that increase proinflammatory responses (Figure 8) [39]. Our observation of greater expression of sPLA_2 _and Msr1 in the spleen of CVB3 infected males may allow oxidation and uptake of LDL-cholesterol and activation of TSPO for the production of steroids in splenic macrophages (Figures 4 and 7). Interestingly, PLA_2 _expression in the heart was found to be a sex-related gene that predicted the progression to heart failure in CVD patients [34]. Haptoglobin was also more highly expressed in the spleen of males prior to infection (Table 2, Figure 2). By binding Apo-AI, haptoglobin inhibits the transfer of lipids from pro-inflammatory LDL particles to anti-inflammatory HDL thereby potentially increasing LDL levels (Figure 8) [39,45].

Findings from our study that examined sex differences in the spleen correspond closely to clinical studies of sex differences in gene expression in CVD and heart failure patients. Sex studies of normal and diseased hearts in rodents and humans have found sex differences in carbonic anhydrase (Table 2 and 3), PLA_2 _(Table 3), Map2k3 (Table 3) and the AR (Figure 3 and 4; see, for example, [4,23,34,37,46]. With an ever increasing world population developing CVD a better understanding of the genes that predispose men to coronary heart disease and DCM is critical. Our findings show that the gene changes that contribute to an increased pro-inflammatory response in males are initiated within hours of infection in the spleen.

## Conclusions

Men are at an increased risk of developing atherosclerosis, myocarditis, DCM and heart failure compared to women but the early immunological factors that drive the proinflammatory response remain unclear. In this study we found that the spleens of CVB3 infected male mice had a greater expression of genes associated with cholesterol and steroid influx and metabolism such as PLA_2_, Msr1 and squalene synthase compared to infected females. TSPO, the rate-limiting step for steroid synthesis and the AR were decreased in CVB3 infected males compared to infected females consistent with activation of the receptors. Thus, increased cholesterol metabolism in the spleen of males during the innate immune response to CVB3 infection may drive the testosterone-mediated proinflammatory response that we observe during acute CVB3 myocarditis, including mast cell and macrophage proliferation, NFκB activation and TLR4 signalling. These findings have important implications for other inflammatory CVDs that are increased in males.

## Abbreviations

AR: androgen receptor; CVB3: coxsackievirus; CVD: cardiovascular disease; DCM: myocarditis and dilated cardiomyopathy; FDR: false discover rate; HPRT: hypoxanthine phosphoribosyl transferase; ip: intraperitoneally; IPA: Ingenuity Pathway Analysis; LDL: low-density lipoprotein; LSD: lest significant difference; LV: left ventricular; PBS: phosphate buffered saline; pi: post infection; PFU: plaque forming units; PLA_2 _: phospholipase A_2_; q RT-PCR: quantitative real-time polymerase chain reaction; TLR: Toll-like receptor; RGF: relative gene expression; TSPO: translocator protein 18 kDa.

## Competing interests

The authors declare that they have no competing interests.

## Authors' contributions

JAO and MJC contributed equally to this study, and are joint first authors. JAO carried out the microarray studies and analysis, participated in qRT-PCR assays and drafted the manuscript. MJC carried out the microarray studies and analysis and the qRT-PCR assays and analysis and helped draft the manuscript. AEG participated in the microarray studies and analysis and in qRT-PCR analysis and conducted plaque assays. JBK conducted plaque assays. AB conducted ELISAs. DB conducted echocardiography and interpreted the results. KLG interpreted the echocardiography results and helped to draft the manuscript. TRG participated in the design of the study and the interpretation of data. DF conceived the study, participated in its design and coordination and helped to draft the manuscript. All authors read and approved the final manuscript.
